# Case Report: Atypical prostate cancer presentation: rectal bleeding, pain, and psoriasiform dermatitis

**DOI:** 10.3389/fonc.2025.1476988

**Published:** 2025-03-26

**Authors:** Frank Obeng, Aishah Fadila Adamu, Samuel Edudzi Gavor, Blessings Yao Setsoafia, Ebenezer Kwame Antwi, Nelson Affram, Ayamba Mamudu Ali

**Affiliations:** ^1^ School of Medicine, Faculty of Surgery, University of Health and Allied Sciences, Ho, Ghana; ^2^ Department of Surgery, Ho Teaching Hospital, Ho, Ghana

**Keywords:** atypical presentation of prostate cancer, lower gastrointestinal bleeding, psoriasiform dermatitis, metastasis, androgen deprivation therapy, gleason score, multidisciplinary approach, case report

## Abstract

Prostate cancer is typically asymptomatic and usually diagnosed through concerted screening programs. However, in settings where there are no existing national prostate cancer screening programs, it may be picked up at the clinics in patients presenting with urinary symptoms, erectile dysfunction, and hematospermia/hematuria. Rare atypical presentations may also occur, delaying diagnosis and management. This case report discusses a 61-year-old male of Black-African descent, whose first presentation to the hospital for a condition ultimately diagnosed as metastatic prostate cancer, was due to lower gastrointestinal bleeding, rectal pain, and psoriasiform dermatitis. The patient’s clinical findings included a moderate-sized (grade 2) nodular prostate on digital rectal examination (DRE), a total serum prostate-specific antigen (PSA) level of >200 ng/mL, low back bone pain, and osteoblastic lesions on lumbosacral spine X-ray. Prostate core biopsy histopathologically confirmed adenocarcinoma with a Gleason score of 4 + 4 = 8. Histopathological examination of the synchronous skin lesions revealed psoriasiform dermatitis. The patient was managed with surgical androgen deprivation therapy (ADT), followed by oral bicalutamide, dermatologist consultation, and blood transfusions. He was also scheduled for further radiotherapy and chemotherapy (to complete the multimodality prostate cancer treatment). This case highlights the importance of considering prostate cancer with atypical presentations and underscores the need for a multidisciplinary approach in managing advanced cases.

## Introduction

**Figure 1:**
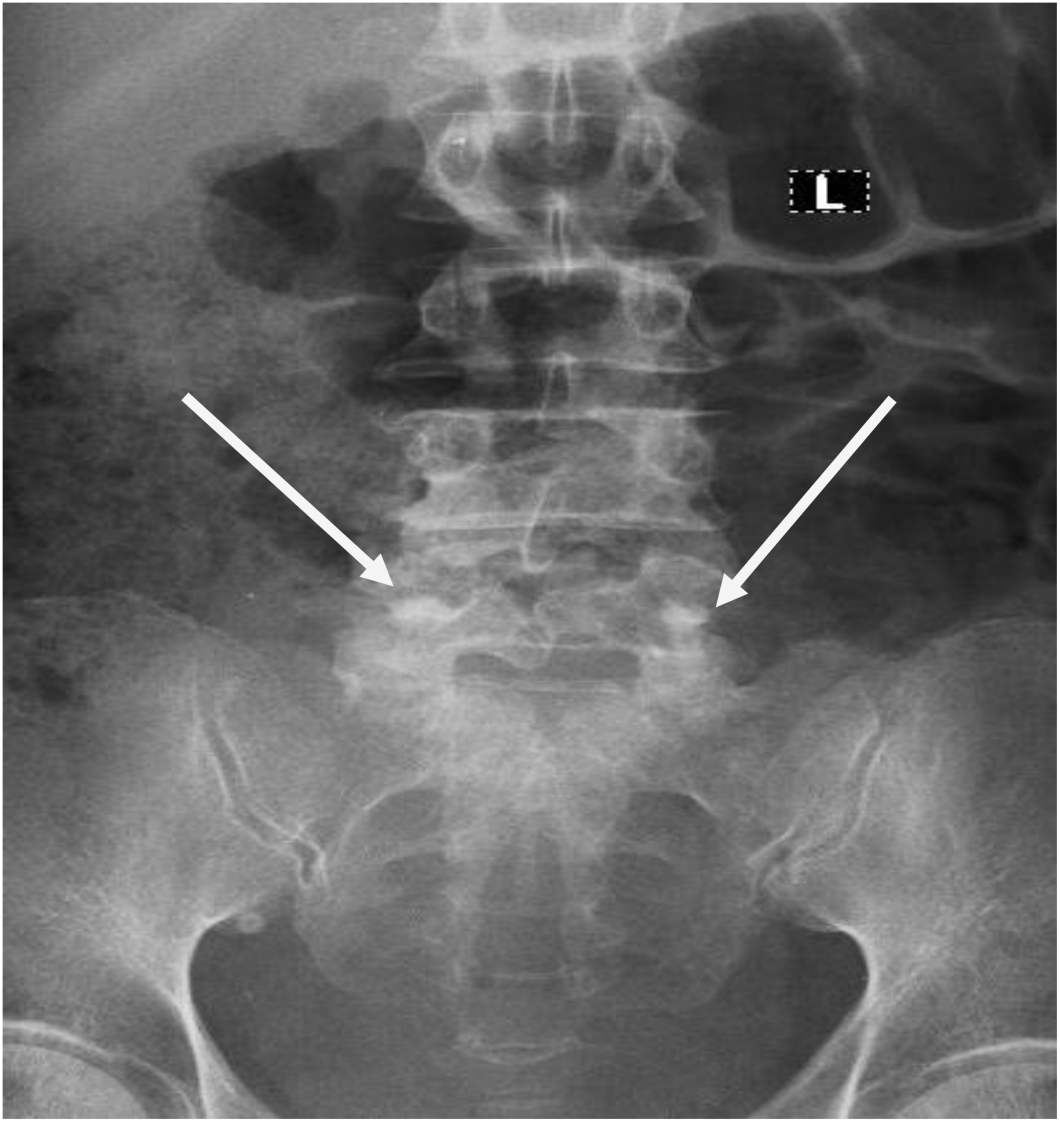
Lumbosacral Spine X-rays; AP-view. Shows suspiscious osteoblastic lesions on L5 (white arrows).

Prostate cancer is the second most common cancer among men worldwide (1). Early prostate cancer is typically asymptomatic and screen-detected but may present with urinary symptoms such as frequency, urgency, hesitancy, erectile dysfunction, and hematospermia/hematuria (2, 3). However, atypical presentations can occur, delaying its diagnosis and management (4–6). This situation may be starker in settings such as Ghana, where there is no concerted national prostate cancer screening program. This case report discusses a 61-year-old male of Black-African descent who presented with lower gastrointestinal (GI) bleeding and an associated psoriasiform dermatitis, ultimately diagnosed with metastatic prostate cancer (**Figure 1**). This case highlights the importance of considering prostate cancer in the differential diagnosis of atypical rectal bleeding.

## Case presentation

### Patient information

The patient was a 61-year-old Black-African male who presented with a one-month history of recurrent bloody stools and a one-week history of dizziness and easy fatigability. He had been experiencing lower urinary tract symptoms (LUTS), including dysuria, frequency, nocturia, and straining, since March 2019, with a worst International Prostate Symptom Score (IPSS) of 14 and a Quality of Life (QOL) score of 5/6.

In February 2022, he sought treatment at an herbal clinic, where he was managed for benign prostatic hyperplasia (BPH) and was started on tamsulosin and finasteride. Despite this, his symptoms progressed, and in June 2023, he developed acute urinary retention requiring the placement of a urethral catheter, which remained *in situ* at the time of his first presentation to the teaching hospital (**Table 1**).

**Table 1:** Timeline of clinical events in the index patient with atypical presentation of metastatic prostate cancer.

Timeline	Event
March 2019	Patient developed dysuria and worsening LUTS (frequency, nocturia, straining). Worst IPSS score = 14, QOL = 5/6.
February 2022	Visited a herbal clinic and was managed as a benign disease.
June 2023	Developed urinary retention and had a urethral catheter passed.
May 2024	First episode of rectal bleeding, which became recurrent. Rectal bleeding started two months after he had started experiencing rectal pain
June 2024	First hospital presentation; developed skin rashes in the nuchal region and back.
July 2024	Admitted to hospital due to anemia and weakness secondary to suspected metastatic prostate cancer. Underwent blood transfusions, colonoscopy, prostate core biopsy, and skin lesion biopsies for diagnosis. Surgical ADT was performed after counseling. Bicalutamide was initiated to bridge the gap before completing counseling for surgical ADT.
November 2024	Skin rash resolved. Follow-up continues
February 2025	Follow-up: Patient is doing well.

LUTS, lower urinary tract symptoms; IPSS score, International Prostate Symptoms Score; QOL, Quality of Life score.

**Figure 2:**
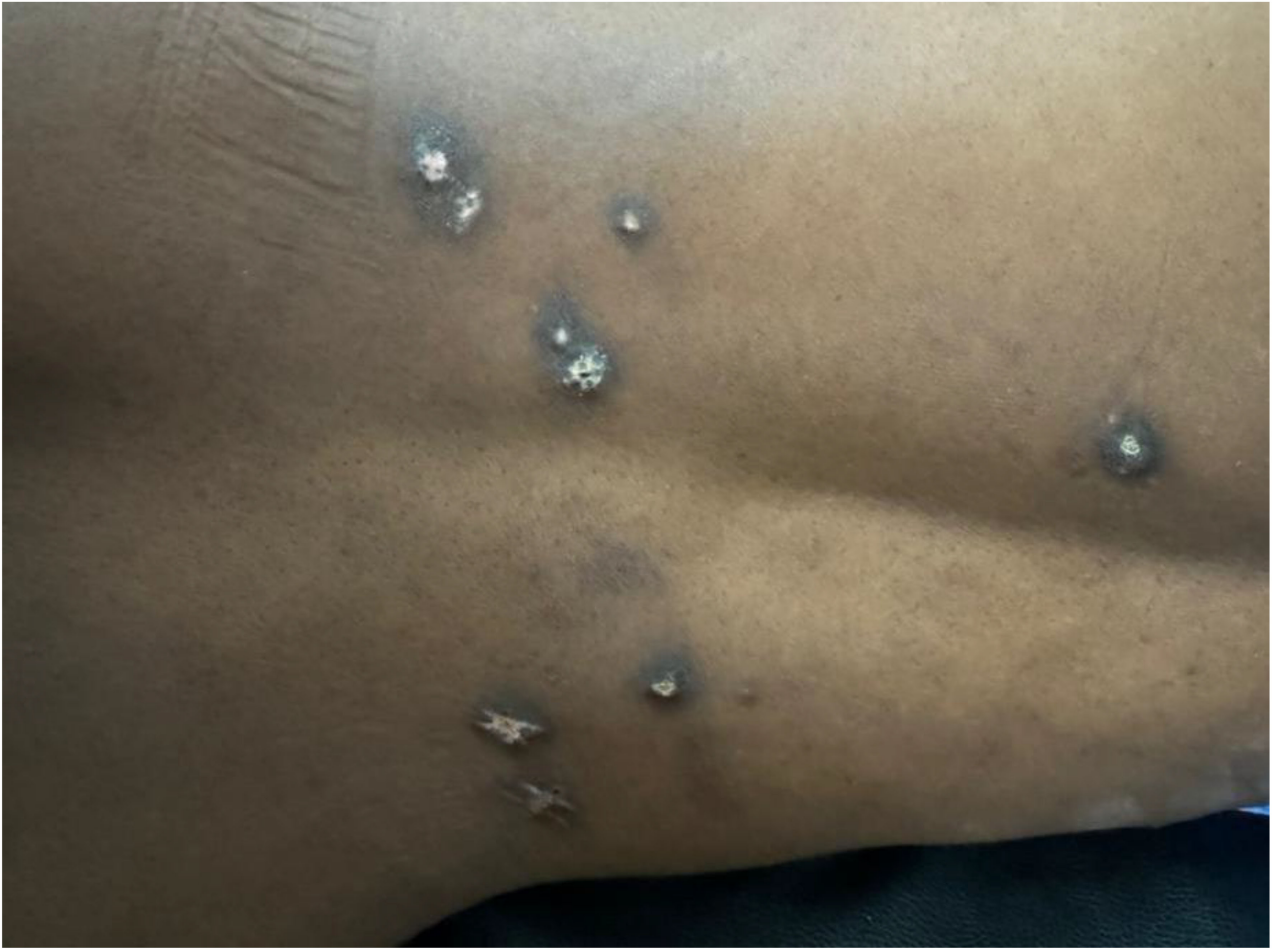
The skin lesions; clinical pictograph. Found to be psoriasiform dermatitis on histopathology of excision biopsies of sampled ones. carcinoma en-cuirasse ruled out.

**Figure 3:**
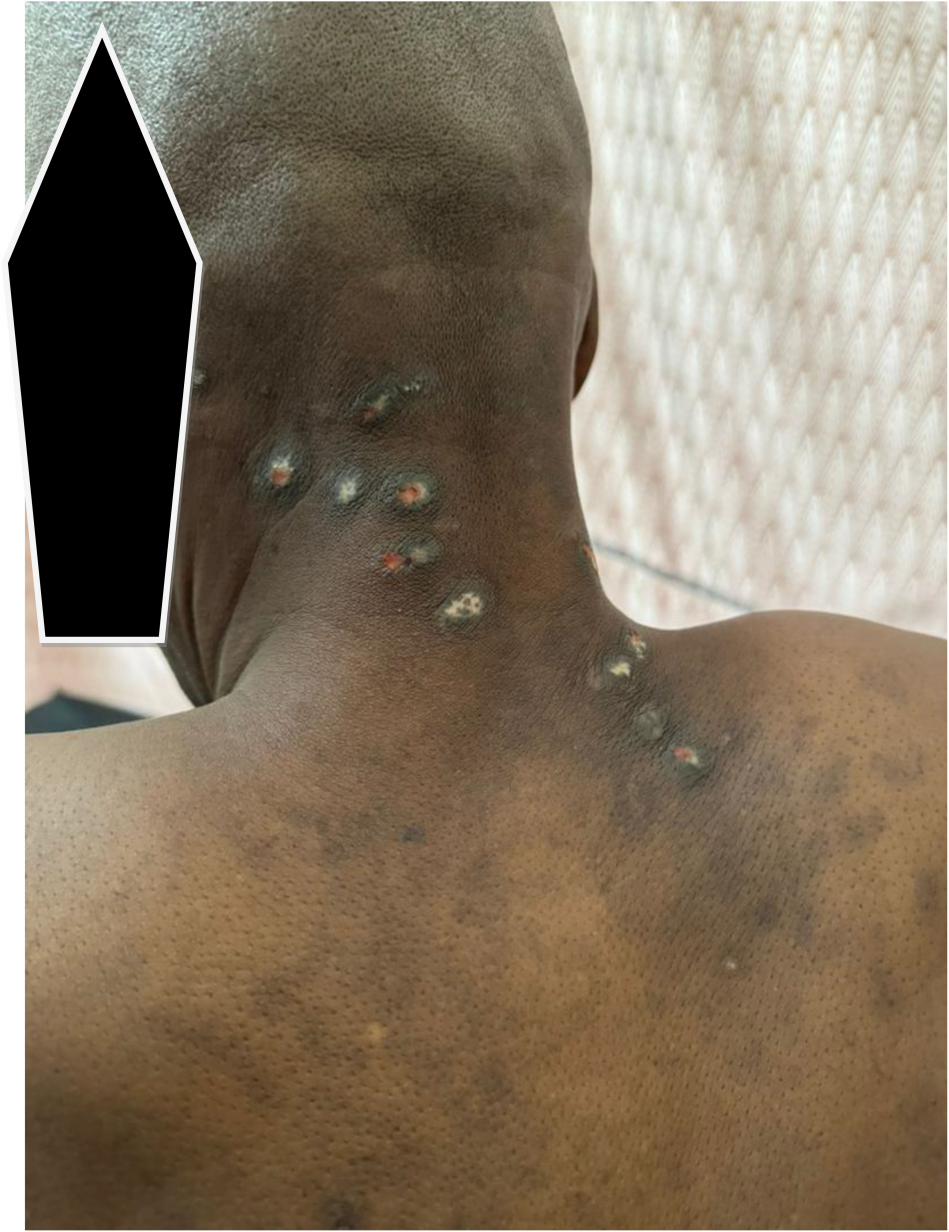
The skin lesions; clinical pictograph of the upper back and nuchal regions. Found to be psoriasiform dermatitis on histopathology of excision biopsies of sampled ones. carcinoma en-cuirasse ruled out.

His symptoms worsened in May 2024 when he experienced his first episode of rectal bleeding with associated rectal pain, both of which recurred. By June 2024, he had developed skin rashes in the nuchal region and back (**Figures 2**, **3**). He presented to the teaching hospital in the same month, where he was found to be anemic and weak, leading to his admission in July 2024 (**Table 1**).

His medical history included hypertension for which he was taking amlodipine 10 mg once daily. His father had a significant family history of prostate cancer, suggesting a genetic predisposition.

### Clinical findings

Upon admission, the patient appeared acutely ill, but not in distress. He had a urethral catheter *in situ* draining amber urine, and his abdomen was flat, with multiple hyperpigmented skin lesions (**Figures 2**, **3**). Digital rectal examination (DRE) revealed a moderately enlarged (grade 2—size), nodular, asymmetrical prostate. His vital signs were stable and he showed no signs of respiratory distress or edema.

### Diagnostic assessment

Initial laboratory investigations revealed anemia (Hb 8.1 g/dL), leukocytosis (WBC 12.27 × 10^9^/L), elevated creatinine (140.18 µmol/L), and a PSA level >200 ng/mL. Imaging studies included a lumbosacral spine X-ray (**Figure 1**) showing osteoblastic lesions at L5, an abdominopelvic ultrasound indicating hepatomegaly (but no nodules), right hydroureteronephrosis, and an enlarged prostate with irregular margins. Chest X-rays revealed no cannonball lesions in the lungs. No other tumor markers, apart from PSA, were assayed.

Proctosigmoidoscopy revealed a nodular prostate with eroded mucosa (over approximately 40% of the prostatic-rectal mucosa coverage area). Under the same proctosigmoidoscopy, a conservative six-core prostate biopsy was performed from the areas of the prostate with apparently healthy/intact rectal mucosal coverage. Histopathology confirmed adenocarcinoma of the prostate, Gleason score 4 + 4 = 8 (acinar, not otherwise specified) with 100% core involvement. Additional biopsy of the skin lesions revealed benign psoriasiform dermatitis.

For further metastatic assessment, the patient was prepared for advanced imaging studies, including a technetium bone scan, the gold standard for bone metastasis evaluation (7). However, this was delayed owing to limited availability.

### Therapeutic intervention

The patient was managed with surgical androgen deprivation therapy (ADT) following a confirmed diagnosis of metastatic prostate cancer. Prior to bilateral orchidectomy, bicalutamide was initiated to provide a temporary androgen blockade. His medications included amlodipine 10 mg once daily (OD) for hypertension, vitamin K 10 mg intramuscularly (IM) daily for five days for coagulopathy correction, and levofloxacin 500 mg OD for infection prophylaxis.

Owing to significant anemia, he received multiple blood transfusions, which led to clinical stabilization.

Given his persistent skin lesions, he was scheduled for dermatological review, and as part of his multimodal treatment plan, he was scheduled to receive external beam radiotherapy and docetaxel-based chemotherapy for advanced metastatic prostate cancer (8).

### Follow-up and outcomes

Following treatment, the patient’s rectal bleeding abated and he demonstrated clinical improvement. His serum total PSA remained >200 ng/mL, while his serum testosterone dropped to 0.2 nmol/L within one-month post-orchidectomy. Hydroureteronephrosis resolved after androgen deprivation therapy (ADT), and his renal function tests were normalized. The catheter was also removed, and he successfully voided per urethra.

He was scheduled for dermatology review due to persistent skin lesions and was scheduled for external beam radiotherapy and docetaxel-based chemotherapy as part of his multimodal treatment for advanced metastatic prostate cancer (8). However, his skin lesions completely disappeared within four months of ADT and dermatological consultation.

Regarding treatment adherence and tolerability, the patient remained compliant with ADT and tolerated bicalutamide and subsequent interventions well with no reported significant adverse effects. No unanticipated complications were noted during the follow-up.

By November 2024, his skin rashes had fully resolved, and at his February 2025 follow-up, he remained clinically stable with no new symptoms.

### Patient perspective

The patient expressed satisfaction with the treatment received, particularly relief from rectal bleeding, improvement in urinary symptoms, and resolution of skin lesions. He acknowledged the challenges faced during his diagnostic journey, including the delayed presentation and diagnosis, but was grateful for the multidisciplinary approach that ultimately led to his diagnosis and management.

He reported feeling physically stronger and more hopeful about his condition after surgical androgen deprivation therapy and supportive care. He also expressed a strong commitment to adhering to follow-up appointments and ongoing treatment plans, recognizing the importance of regular monitoring and early intervention to optimize health outcomes.

## Discussion

Prostate cancer typically presents with lower urinary tract symptoms (2, 9); however, atypical presentations (6), such as lower GI bleeding, can occur, as seen in the index case. This can be attributed to the invasion of the rectal mucosa by locally advanced tumors, a rare but documented phenomenon (5, 10). The presence of psoriasiform dermatitis in this case adds another layer of complexity, as skin metastasis from prostate cancer, although rare, has been reported previously (11).

The diagnosis of metastatic prostate cancer in the index case was supported by high PSA levels, histopathological findings (which confirmed the cancer), and imaging studies indicating osteoblastic lesions. Management includes hormonal therapy, surgical intervention, and planning for radiotherapy and chemotherapy, reflecting a comprehensive approach for advanced prostate cancer (12).

The presentation of prostate carcinoma with lower gastrointestinal (GI) bleeding is atypical. Prostate cancer is generally associated with urinary symptoms, bone pain, and sometimes systemic manifestations (3) but rarely with rectal bleeding. In this case, SD, a 61-year-old male, presented with rectal bleeding, dizziness, and easy fatigability, which led to the discovery of locally advanced likely metastatic prostate cancer. This discussion explores the unusual presentation, diagnostic challenges, and therapeutic strategies involved in the management of this case.

### Atypical presentation

Prostate cancer typically manifests with urinary symptoms, such as frequency, urgency, nocturia (2), and hematuria. Metastasis often involves the bones, leading to bone pain and fractures (13). However, rectal bleeding as a presenting symptom is rare and can lead to misdiagnoses. The patient’s symptoms were initially suggestive of primary colorectal pathology. Rectal bleeding in the patient is likely due to direct invasion of the rectal mucosa by the prostate tumor, which underscores the aggressive nature of the disease (14). Prostate cancer invading the rectum is rare, but has been documented in advanced cases, suggesting a poor prognosis and a higher likelihood of metastatic spread (10).

### Diagnostic challenges

Initial clinical evaluation and imaging studies, including colonoscopy, are crucial in excluding primary colorectal malignancies. A nodular, irregular prostate with a PSA level exceeding 200 ng/ml was strongly indicative of advanced prostate cancer. The diagnosis was further supported by histopathological examination of the prostate biopsy specimen, which revealed a Gleason score of 8 (4 + 4) adenocarcinoma with extensive involvement.

The presence of osteoblastic lesions in the lumbosacral spine on X-ray and high PSA levels were consistent with metastatic prostate cancer. Osteoblastic lesions are hallmarks of prostate cancer metastasis to the bone and are characterized by the formation of new bone material (15). The patient’s anemia was likely multifactorial, resulting from chronic disease and possibly from ongoing blood loss (14).

### Psoriasiform dermatitis

Another atypical aspect of the patient’s presentation was the presence of psoriasiform dermatitis, a condition that is not commonly associated with prostate cancer. Skin manifestations in prostate cancer are rare and typically associated with paraneoplastic syndromes or direct metastatic involvement (16). The histopathology of the skin lesions revealed (a benign condition) psoriasiform dermatitis rather than metastatic disease, adding another layer of complexity to his clinical picture. Psoriasiform dermatitis in the context of cancer may be related to immune dysregulation, although its exact association with prostate cancer remains unclear (16, 17).

Prostate cancer, especially in the advanced stages, can lead to immunological dysregulation. This dysregulation can result from cancer itself and the body’s response to cancer. Importantly, even in the absence of treatment, cancer can cause significant changes in immune function (18).

### Immunological dysregulation in advanced prostate cancer

1. Cancer and Immune System Interaction: Advanced prostate cancer can affect the immune system in several ways. Tumor cells can evade the immune system through various mechanisms, including alteration of the tumor microenvironment and suppression of the immune response. Chronic inflammation associated with cancer can also lead to immune system changes (18).
2. Treatment-Induced Dysregulation: Treatments for advanced prostate cancer, such as chemotherapy, androgen deprivation therapy (ADT), and newer immunotherapies, can also affect the immune system. These treatments may weaken the immune response or, conversely, trigger immune-related adverse events (18, 19).

### Skin manifestations

1. Paraneoplastic Syndromes: Advanced cancers, including prostate cancer, occasionally lead to paraneoplastic syndromes. These conditions are caused by the immune system’s response to the tumor and can affect various organs, including the skin (16).
2. Generalized Psoriasiform Dermatitis: Although less common, advanced prostate cancer is associated with paraneoplastic skin conditions. Psoriasiform dermatitis, which is characterized by skin lesions resembling psoriasis, can occur in patients with paraneoplastic syndromes. This type of dermatitis is marked by red scaly patches that can spread across the body (16, 20).

Mechanisms: The exact mechanisms by which prostate cancer might lead to generalized psoriasiform dermatitis are not fully understood but could involve the following:

- Cytokine release and inflammatory mediators from tumor and immune cells (18).
- Cross-reactivity of immune cells, targeting both tumor and skin antigens (16).
- Immune system dysregulation induced by cancer (18).

In this case, the patient developed generalized psoriasiform dermatitis before initiating treatment for prostate cancer. This suggests that immunological dysregulation and the resulting skin lesions were related to the cancer itself, rather than a side effect of cancer treatment. This aligns with the understanding that advanced prostate cancer can cause significant immune system changes independent of external therapeutic interventions (18).

In conclusion, advanced prostate cancer can cause immunological dysregulation, leading to various systemic manifestations, including skin conditions, such as generalized psoriasiform dermatitis. If a patient with advanced prostate cancer presents with unusual or widespread skin lesions, it is essential to consider the potential paraneoplastic nature of these symptoms and to manage them appropriately in conjunction with oncological treatment.

### Multidisciplinary management

Patient management required a multidisciplinary approach involving urology, proctology, oncology, and dermatology. The initial therapeutic strategy focused on controlling bleeding and addressing metastatic disease. Androgen deprivation therapy (ADT) with Casodex (bicalutamide) *a priori*, followed by surgical castration, is a common first-line treatment for advanced prostate cancer (21–23). ADT aims to reduce the levels of androgens that fuel prostate cancer growth (22). In addition to hormonal therapy, the anemia was managed with blood transfusions.

### Future therapeutic considerations

Given the index case’s high-risk and advanced metastatic prostate cancer, his treatment plan included potential external beam radiotherapy and chemotherapy with docetaxel, which are standard treatments for advanced prostate cancer with bone metastases. Studies have shown that combining ADT with radiotherapy and chemotherapy can improve survival outcomes in patients with metastatic hormone-sensitive prostate cancer (12).

Furthermore, this case highlights the importance of considering atypical presentations in patients with known risk factors for prostate cancer. The patient’s positive family history of prostate cancer and prior diagnosis of BPH should have heightened clinical suspicion earlier in his presentation. This underscores the need for comprehensive evaluation and a high index of suspicion in similar cases (24, 25).

### Conclusion

This case illustrates the diagnostic challenges of atypical prostate cancer presentations such as rectal bleeding, rectal pain, and psoriasiform dermatitis. The modest success of therapy in this advanced stage of diagnosis, further emphasizes the need for early recognition and multidisciplinary approaches for prostate cancer management.

Further research is essential to understand these atypical manifestations and improve early detection and management strategies.

## Data Availability

The original contributions presented in the study are included in the article/supplementary material. Further inquiries can be directed to the corresponding author.
